# High Resistance of *Plasmodium falciparum* to Sulphadoxine/Pyrimethamine in Northern Tanzania and the Emergence of dhps Resistance Mutation at Codon 581

**DOI:** 10.1371/journal.pone.0004569

**Published:** 2009-02-24

**Authors:** Samwel Gesase, Roly D. Gosling, Ramadhan Hashim, Rosalynn Ord, Inbarani Naidoo, Rashid Madebe, Jacklin F. Mosha, Angel Joho, Victor Mandia, Hedwiga Mrema, Ephraim Mapunda, Zacharia Savael, Martha Lemnge, Frank W. Mosha, Brian Greenwood, Cally Roper, Daniel Chandramohan

**Affiliations:** 1 National Institute for Medical Research, Tanga Centre, Tanga, Tanzania; 2 Department of Infectious and Tropical Diseases, London School of Hygiene and Tropical Medicine, London, United Kingdom; 3 Malaria Research Lead Programme, Medical Research Council, Durban, South Africa; 4 Kilimanjaro Christian Medical College, Moshi, Tanzani; University of California Los Angeles, United States of America

## Abstract

**Background:**

Sulphadoxine-pyrimethamine (SP) a widely used treatment for uncomplicated malaria and recommended for intermittent preventive treatment of malaria in pregnancy, is being investigated for intermittent preventive treatment of malaria in infants (IPTi). High levels of drug resistance to SP have been reported from north-eastern Tanzania associated with mutations in parasite genes. This study compared the *in vivo* efficacy of SP in symptomatic 6–59 month children with uncomplicated malaria and in asymptomatic 2–10 month old infants.

**Methodology and Principal Findings:**

An open label single arm (SP) standard 28 day *in vivo* WHO antimalarial efficacy protocol was used in 6 to 59 months old symptomatic children and a modified protocol used in 2 to 10 months old asymptomatic infants. Enrolment was stopped early (87 in the symptomatic and 25 in the asymptomatic studies) due to the high failure rate. Molecular markers were examined for recrudescence, re-infection and markers of drug resistance and a review of literature of studies looking for the 581G dhps mutation was carried out. In symptomatic children PCR-corrected early treatment failure was 38.8% (95% CI 26.8–50.8) and total failures by day 28 were 82.2% (95% CI 72.5–92.0). There was no significant difference in treatment failures between asymptomatic and symptomatic children. 96% of samples carried parasites with mutations at codons 51, 59 and 108 in the *dhfr* gene and 63% carried a double mutation at codons 437 and 540. 55% carried a third mutation with the addition of a mutation at codon 581 in the *dhps* gene. This triple: triple haplotype maybe associated with earlier treatment failure.

**Conclusion:**

In northern Tanzania SP is a failed drug for treatment and its utility for prophylaxis is doubtful. The study found a new combination of parasite mutations that maybe associated with increased and earlier failure.

**Trial Registration:**

ClinicalTrials.gov NCT00361114

## Introduction

Sulphadoxine-pyrimethamine (SP) is one of the most widely used antimalarials worldwide. It is used as first line treatment for uncomplicated malaria alone or in combination with other antimalarials, although it has been replaced with other antimalarials in Southeast Asia and sub-Sahran Africa because of high levels of resistance. SP is also recommended for use as Intermittent Preventive Treatment of malaria in pregnancy (IPTp)[1] in sub-Saharan Africa and is currently being investigated for use as Intermittent Preventive Treatment of malaria in infants (IPTi)[2]. SP has been used in North-eastern Tanzania since the early nineteen nineties[3] and was adopted as the first line antimalarial for uncomplicated malaria nationally in 2001[4]. *Plasmodium falciparum* resistance to SP has been recorded in Muheza, north-eastern Tanzania since 1994[5], [6] and 1995[7] and when SP was adopted as the first line drug the adequate clinical and parasitological response (ACPR) by day 14 had already fallen to 76% in Tanga region, Tanzania[8]. As part of an ongoing trial of IPTi using SP, mefloquine and chlorproguanil-dapsone in this region, we evaluated the *in vivo* efficacy of SP in clearing parasites (clinical efficacy) in order to understand the relationship between the clinical efficacy and the protective efficacy of SP-IPTi

Traditionally, antimalarial drug efficacy is measured using the standard WHO 28 day *in vivo* protocol[9] in symptomatic, 6–59 month old children. However, results obtained in this population may not be representative of the efficacy of a drug when used for prevention. When used for prevention, antimalarials work both by preventing new blood stage infections, the prophylactic effect, as well as by clearing parasites present in those who are asymptomatic but infected with malaria[10]. Since asymptomatic subjects generally have a lower level of parasitaemia than clinical cases[11] even failing antimalarials may be effective at clearing parasitaemia in asymptomatic subjects as these individuals are likely to have some degree of naturally acquired immunity. Therefore, we have studied the *in vivo* efficacy of SP in both symptomatic 6–59 month old children and asymptomatic 2–10 month old children, the target group for IPTi.

Parasite susceptibility to SP is influenced by mutations in two genes. Resistance to pyrimethamine is determined by point mutations at codons 16, 50, 51, 59, 108 and 164 of the *dhfr* gene[12], [13] and resistance to sulphadoxine by mutations at codons 436, 437, 540, 581 and 613 of the *dhps* gene[14], [15]. In Africa, the presence of three *dhfr* mutations (N51I, C59R, S108N) together with two *dhps* mutations (A437G, K540E) prior to treatment is a significant predictor of SP *P. falciparum* treatment failure[16], [17], [18]. A mutation at codon 164 in the *dhfr* gene became established in south east Asia twenty years ago and was found to be associated with a high level SP resistance, as well as resistance to chlorproguanil-dapsone[19] and artesunate-dapsone-proguanil[20]. 164 mutants have recently begun to emerge in Africa although it is not yet clear that these mutations herald the emergence the same highly resistant phenotype in-vivo[21], [22], [23], [24]. Because of the association of mutations in the *dhfr* and *dhps* genes with resistance to SP we studied, molecular markers in symptomatic children and asymptomatic infants who had parasitological failures.

## Methods

The protocol for this trial and supporting CONSORT checklist are available as supporting information; see Protocol S1 and Checklist S1.

### Study site

The study was conducted in Hale Health Centre, Tanga Region, situated 32 km north of Muheza where SP resistance was first observed in Tanzania. The district experiences perennial, holoendemic malaria although, in recent years, transmission appears to have declined substantially. The entomological inoculation rate from the neighbouring district of Muheza was 148 infectious bites per person per year in 2000[25]. The study site was chosen due to its proximity to the site of a clinical trial of IPTi comparing SP, chlorproguanil-dapsone and mefloquine. The study protocol was approved by the Ethics Review Committees of the National Institute of Medical Research of Tanzania and the London School of Hygiene and Tropical Medicine and was registered as a clinical trial with the National Institute of Health (Clinicaltrials.gov identifier NCT00361114). The protocol included an arm for chlorproguanil/dapsone in the 6–59 month study after the SP arm was completed, however it was not possible to procure the drug and recently the drug has been withdrawn from clinical development[26].

### Participants

#### Symptomatic study in children aged 6–59 months

All children between the ages 6 and 59 months who attended Hale Health Centre during July–August 2006 with a fever or history of fever during the study period were screened for malaria using a rapid diagnostic test (RDT) (Paracheck, Orchid Biomedical Systems, Verna, India). Children with a positive RDT had a thick blood smear read and those with a positive blood smear were referred to the study clinician. Study inclusion criteria were: (1) weight of ≥4.5 kgs, (2) not -enrolled in the IPTi trial, (3) absence of severe malnutrition (weight-for-height <3 standard deviations from the norm), (4) slide-confirmed infection with *P. falciparum* only with an initial parasite density of between 2,000 and 200,000 asexual parasites per microliter, (5) absence of general danger signs (inability to drink or breastfeed; vomiting; recent history of convulsions; lethargy or unconsciousness; inability to sit or stand up) or other signs of severe and complicated falciparum malaria according to WHO definitions, (6) measured axillary temperature ≥37.5°C, (7) ability to attend stipulated follow-up visits, (8) informed consent provided by parent/guardian; (9) absence of history of hypersensitivity reactions to SP and (10) no prior antimalarial use in the preceding 2 weeks.

#### Asymptomatic study in children aged between 2 and 10 months

Consent was obtained from caretakers of 2–10 month old infants who attended the Maternal Child Health (MCH) clinic for immunization or weighing, for screening for *P. falciparum* infection. From those consented for screening, finger prick blood was obtained for the rapid diagnostic test, thick and thin blood smear preparation and filter paper samples for molecular studies. Children who had a positive blood slide were further assessed for their eligibility for inclusion into the study and enrolled in the drug sensitivity study after obtaining an informed consent. The eligibility criteria were the same as for the study of symptomatic children except that there was no history of fever in the last 48 hours, that measured axillary temperature should be less than 37.5°C and that the presence of *P. falciparum* parasitaemia at any density was acceptable.

#### Sample size

In order to detect a 15% difference in adequate parasite clearance by day 28 between symptomatic 6–59 month old children and asymptomatic 2–10 month old children with 80% power at the 5% significance level using a ratio of 2 symptomatic cases to 1 asymptomatic case, we estimated that 292 symptomatic children and 146 asymptomatic infants would be required.

#### Treatment allocation

Children were treated with SP (Fansidar®, Roche, France) by weight (1 tablet containing 500 mg sulfadoxine and 25 mg pyrimethamine; ½ tablet for weights 4.5–10 kg, 1 tablet for 11–20 kg, 1½ tablets for 21–30 kg). The content and solubility of the SP tablets were confirmed by solubility testing and high performance liquid chromatography at the London School of Hygiene and Tropical Medicine. The study drugs achieved the expected concentrations of SP in solution when compared to controls (Fansidar®, Roche, France) purchased in the UK. SP was given under observation by study staff and children were observed for at least 1 hour after treatment. If a child vomited within 30 minutes of receiving the drug, the full dose was repeated. if a child vomited between 30 minutes and an hour, half the dose was repeated. If a study child vomited the study medication twice, the study child was given rescue treatment and excluded from the study. Rescue treatment for uncomplicated malaria was artemether- lumefantrine (Coartem®, Novartis, Basel, Swizterland) and for severe malaria was parenteral quinine.

#### Follow up

Children in the symptomatic study were seen at the clinic on days 1, 2, 3, 7, 14, 21, and 28 after treatment and home visits were made for those who failed to report. Parents were encouraged to bring any child who became ill between specified visits to the clinic where they were evaluated and treated by a study clinician thoughout the study period. Malaria blood films and filter paper samples were obtained from children in the symptomatic group at all active and passive follow-up time points. Blood samples were not collected on days 1, 2, 3, and 21 from the asymptomatic infants if the infant remained well. However, blood samples were collected at any time if the infant became symptomatic.

#### Endpoints

The primary end point, parasitological failure by day 28 was defined as (1) development of danger signs or severe malaria, (2) parasitaemia on Day 2 that was higher than that on Day 0, (3) parasitaemia on Day 3 ≥25% of the count on Day 0, (4) parasitaemia on or after Day 4. Failures were further divided into early treatment failures (within day 3 post treatment), late clinical failures (recorded fever plus parasitaemia from day 4 to day 28 post treatment), and late parasitological failure (parasitaemia at day 14 or day 28 post treatment in the absence of fever). In the symptomatic study children with parasitaemia on or after day 4 were treated with rescue treatment if they became symptomatic or until they reached day 28 when all parasitaemic children were treated. In the asymptomatic study any child on or after day 4 with parasitaemia was treated with rescue treatment.

#### Review of the prevalence of the A581G mutation in Africa

The literature search for reports of the A581G mutation up to October 2007 was done using the National Library of Medicine search engines, Pubmed and Medline. The following terms were included in the search queries: *dhfr*, *dhps*, sulphadoxine, sulfadoxine, pyrimethamine, Fansidar, Africa, prevalence, malaria and resistance. To be included in the review, articles had to include analysis of codon 581 of the dhps gene in isolates collected from study sites in Africa.

#### Laboratory Procedures

Blood smears were stained with 20% Giemsa for 20 minutes and read by two independent microscopists for speciation, and quantification. Parasite density was estimated by counting parasites against 200 White Blood Cells (WBC). A blood smear was considered negative if no asexual forms were seen after observing 500 WBC. Discordant results (33% difference in quantification or positive/negative results) were read by a third microscopist; agreement between any two micoscopists and the average parasite density were deemed to be the correct finding. Parasite counts were adjusted assuming a standard WBC of 8000 per microlitre.

DNA was extracted from bloodspots dried on filter papers by soaking overnight in 1 mL. of 0.5% saponin-1× phosphate buffered saline (PBS). The segment was then washed twice in 1 ml of PBS and boiled for 8 min in 100 µL PCR quality water with 50 µL 20% chelex suspension (pH 9.5). *dhfr* and *dhps* were PCR amplified and point mutations at codons 51, 59, 108 and 164 of the *dhfr* gene and codons 436, 437, 540, 581, and 613 of the *dhps* gene were genotyped using a dotblot methodology previously described by Pearce et al[27]. The probed blots were visualised through alkaline phosphatase-catalysed breakdown of the flourogenic substrate (ECF) (GE Healthcare, Buckinghamshire, UK) and the chemifluorescent signal scanned on a TYPHOON Trio® Phosphoimager (GE Healthcare, Buckinghamshire, UK). The stringency and specificity of the hybridisation process was confirmed by inspection of a series of four controls of known single genotype variant sequence. All blots with non-specifically bound probes were stripped and re-probed. A sequence variant was considered to be present in the PCR product when the intensity of signal was higher than that of the background. The presence, absence, and relative abundance of hybridisation signal was recorded for every probe at each locus. A sample was considered to have a single haplotype when only one sequence variant was found at each locus. Where alternative sequences were present in the same these were designated as a mixed genotype infection. Where mixture was detected at one locus only we inferred a mixture of 2 haplotypes which varied only by that codon.

We tested for the presence of mutations in addition to those at codon 436, 437, 540, 581, and 613 of dhps by direct sequencing. PCR products were purified using ExoSAP-IT® (USB Corporation, Cleveland, Ohio, USA). Cycle sequencing was performed using Applied Biosystems BigDye V 3.1 and samples loaded on the ABI-3730 capillary system. Sequence reads were checked by eye and edited using the Seqman (DNAstar Inc., Madison, WI, USA). The presence of SNPs was confirmed by reads through both forward and reverse strands.

Recrudescent and new infections were differentiated first by typing size and sequence of the highly polymorphic repeat region of MSP2[28] and then by typing repeat length polymorphism at the PfPK2 microsatellite marker[29]. The size polymorphism of PCR amplified FC27 and IC1 fragments of MSP2, was determined by agarose gel electrophoresis, stained with SYBR® Green 1 (Invitrogen™ Ltd, Paisley, UK) and scanned on a TYPHOON Trio® phosphoimager (GE Healthcare, Buckinghamshire, UK). The gel image was analysed using ‘Imagequant software™’(Molecular Dynamics, Foster City, CA, USA) and fragment sizes were calibrated to known fragment sizes in HyperladderIV (Bioline™, London, UK) which was run in duplicate on every gel. Pre- and post-treatment samples for each patient were compared according to sequence and size of the PCR amplified MSP2 fragments. Recrudescent infections were characterized as having at least one identical allele present in both pre and post treatment samples. Matching alleles were defined as those for which the analysis software estimated the sizes to be within 15 bp of each other. Samples where no alleles matched in the pre and post treatment were classified as new infections. Pre-and post treatment sample pairs which were classified as having a recrudescent infection according to MSP2 matches were then compared at the Pfpk2 microsatellite locus. The Pfpk2 microsattelite repeat was pcr amplified using the protocol described in Anderson et al[29] and fragments were run on an ABi 3730 DNA analyzer (Applied Biosystems, Foster City, USA) with LIZ-500 size standard and analyzed using Genemapper software (Applied Biosystems, Foster City, USA). Any pairs of pre and post treatment samples which matched at MSP2 but did not match at PfPK2 were re-classified as reinfections. If either pre or post sample failed to amplify, they were classified as undetermined.

Multiplicity of infection (MoI) was assessed by examining the numbers of alleles detected at MSP2 and pfPK2. Where the number of alleles at these two loci differed, the higher of the two values was used since this is the minimum number co-infecting genotypes which can explain the observed diversity.

#### Statistical analysis

Data were double entered into an Access (Microsoft Corps, Seattle, USA) data base and analyzed in STATA 9.0 (Stata Corps,Texas, USA). Crude and PCR-corrected rates (excluding new infections and indeterminate PCR) were estimated. A survival analysis[30] was carried out by censoring children at the time of a PCR-corrected new infection or undetermined treatment failure and loss to follow up.

Logistic regression was used to determine factors associated with treatment failures (cases) compared to non-failures (controls). The following variables identified *a priori* were included in the logistic regression model: age, parasite density, study population (asymptomatic or symptomatic) and molecular markers. In addition, any factor that was significant at the 10% level in the crude analysis was included in the model.

Further survival and regression analyses were carried out in order to examine the relationship with time to failure and the presence of the mutation 581G in the *dhps* gene.

## Results

### Response of symptomatic children and asymptomatic infants to treatment with SP

**Figure 1:**
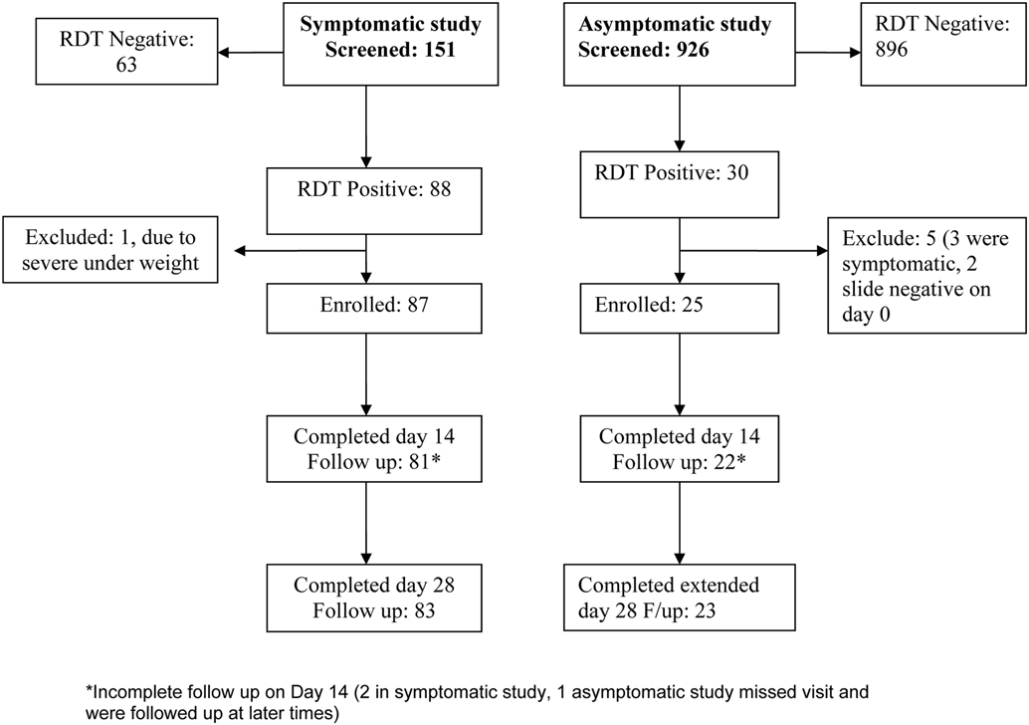
Trial profiles. *Incomplete follow up on Day 14 (2 in symptomatic study, 1 asymptomatic study missed visit and were followed up at later times).

One hundred and fifty, 6–59 month-old febrile children were screened for malaria in July–August 2006 and 87 children who fulfilled the inclusion criteria were enrolled (see figure 1). Recruitment to the study arm was stopped early when the study team observed an unacceptably high failure rate and 3 children progressed to severe disease within 3 days post treatment with SP, including one death due to severe malarial anaemia and respiratory failure. No other adverse events were reported.

Between October 2006 and June 2007, 926 asymptomatic 2–10 month-old infants were screened for malaria and 25 infants with parasitaemia were enrolled into the study arm. This study was also stopped early due to a high failure rate with one child progressing to severe disease within 3 days post treatment. Apart from this serious adverse event no other adverse events were reported. The trial profiles are shown in figure 1. As expected, the two study populations differed significantly in age, weight, temperature and parasite density but not in mean haemoglobin concentration (Table 1).

**Table 1:** Baseline characteristics of the two study populations.

Parameter	Symptomatic children (N = 87)	Asymptomatic infants (N = 25)	P value
Mean age (months, SD)	31.1 (13.3)	7.1 (2.2)	<0.001
Mean weight (Kg, SD)	11.9 (2.3)	7.6(1.0)	<0.001
Mean haemoglobin (g/dl, SD)	9.04 (1.9)	8.6 (1.1)	0.353
Mean temperature (°C, SD)	38.5 (3.2)	36.7 (0.32)	0.005
Temperate Range (°C)	37.5–40.9	36.0–37.5	<0.001
Geometric mean density (asexual forms/µL)	13,914	4,173	<0.001

**Figure 2:**
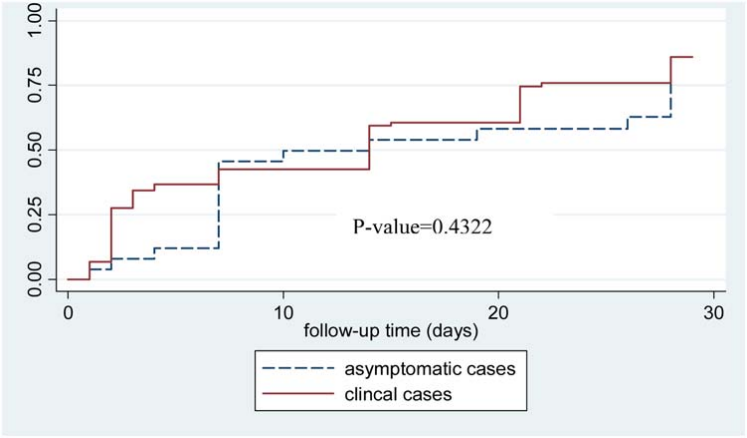
Cumulative proportion of treatment failures of Sulphadoxine-Pyrimethamine in symptomatic 6–59 month and asymptomatic 2–10 month old children with malaria.

The therapeutic efficacy of SP by day 14 and 28 post treatment is shown in Table 2. There was no statistically significant difference in failure rates corrected by PCR for new infections between symptomatic and asymptomatic groups by Day 14 (39% vs 53%; p = 0.37) or Day 28 (82% vs 77%; p = 0.35). The survival analysis (Figure 2) shows there was no significant difference between PCR uncorrected results for the symptomatic and asymptomatic groups in the time to treatment failure. When adjusted for the effect of age, sex and parasite density at enrolment, the risk of treatment failure was slightly higher in the symptomatic children compared to the asymptomatic infants but this was not statistically significant (odds ratio 1.2; 95% CI 0.55, 2.9; p = 0.6). Early Treatment Failures were less common and Late Parasitological Failures were more common in the asymptomatic group compared to symptomatic children (table 2). There was no statistical difference between the prevalence of new infections in either group (23% vs 11%, symptomatic and asymptomatic groups respectively, p = 0.6).

**Table 2:** *In vivo* efficacy of Sulphadoxine-pyrimethamine in symptomatic 6–59 month and asymptomatic 2–10 month old children.

Outcome	Uncorrected		P-value	PCR Corrected		P-value
Day 14	Symptomatic(N = 81)	Asymptomatic(N = 22)		Symptomatic(N = 67+)	Asymptomatic(N = 17*)	
	% (95% CI)	% (95% CI)		% (95%)	% (95%)	
ETF	37.0 (26.3–47.8)	9.1(−3.9–22.1)	0.012	38.8 (26.8–50.8)	5.9 (−6.6–18.3)	0.009
LCF	27.2 (17.3–37.1)	18.2 (0.7–35.7)	0.390	22.4(12.1–32.6)	23.5 (1.0–46.0)	0.920
LPF	3.7 (−0.5–7.9)	31.8 (10.7–52.9)	<0.001	-	17.6 (−2.5–37.8)	-
**Clinical or parasitological failure by day 14**	**67.9 (57.0–78.0)**	**59.1 (36.7–81.4)**	**0.439**	**61.2(49.2–73.2)**	**47.1(20.6–73.5)**	**0.291**
ACPR	32.1 (21.7–42.5)	40.9 (18.6–63.2)	0.439	38.8 (26.8–50.8)	52.9(26.5–79.4)	0.291
**Day 28**	**(N = 83)**	**(N = 23)**		**(N = 62** ++ **)**	**(N = 17** ** **)**	
ETF	36.1 (25.6–46.7)	8.7 (−3.8–21.1)	0.011	41.9 (29.3–54.6)	5.9 (−6.6–18.3)	0.005
LCF	42.2 (31.3–46.7)	26.1 (6.7–45.5)	0.161	37.1 (24.7–49.5)	35.3 (9.9–60.6)	0.891
LPF	8.4 (2.3–14.5)	47.8 (25.7–69.9)	<0.001	3.2 (0.77–11.3)	35.3 (9.9–60.6)	<0.001
**Clinical or parasitological failure by day 28**	**86.8 (63.1–96.1)**	**82.6 (79.3–94.2)**	**0.614**	**82.2(72.5–92.0)**	**76.5 (49.3–95.1)**	**0.590**
ACPR	13.2 (5.8–20.7)	17.4(0.6–34.1)	0.614	17.7 (8.0–27.5)	23.5 (1.0–46.0)	0.590

+ 9 had new infection, 5 had PCR results missing.

++ 19 had new infection, 2 had PCR results missing.

* 2 had new infection, 3 had PCR results missing.

** 2 had new infection, 4 had PCR results missing.

ETF : Early Treatment Failure.

LCF: Late Clinical Failure.

LPF: Late Parasitological Failure.

ACPR: Adequate Clinical and Parasitological Response.

### Molecular findings

**Figure 3:**
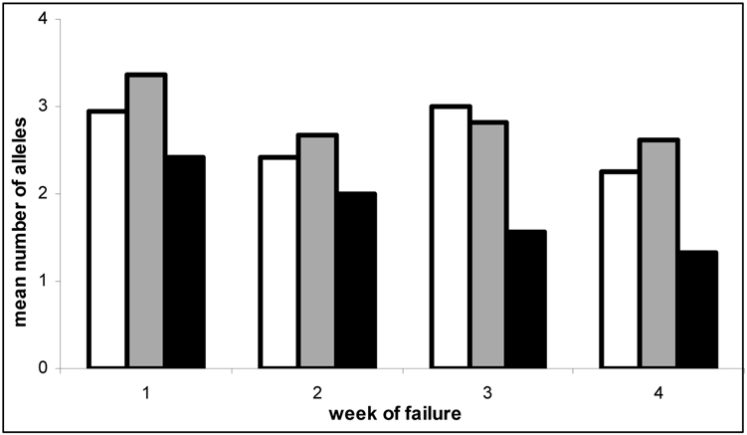
Comparison of Multiplicity of Infection (MoI) at enrolment and at day of failure and the mean MoI in cases of recrudescence by week of failure. LEGEND: 67 symptomatic treatment failures cases were successfully analysed by both MSP2 and pfPK2 and were sorted according to week of failure post treatment. White bars show the mean multiplicity of infection (MoI) at enrolment. Grey bars show the MoI on the day of treatment failure. Black bars show the mean number of matching alleles in the pre and post treatment sample of each patient (N = 49 after exclusion of those defined as new infections).

New infections were detected at all time periods in the study. In the symptomatic group 4, 5 and 10 new infections were detected between days 1–3, 4–14 and 15–28 respectively and in the asymptomatic group 2 new infections were detected between days 4 and 14. Molecular typing revealed a high rate of multiple clone infection both pre and post treatment, with between 1–4 MSP2 alleles and 1–8 pfPK2 alleles found in individual infections. Figure 3 shows the multiplicity of infection (MoI) by showing the mean number of parasite clones present at enrolment, on the day of failure and the mean number of clones in cases of recrudescence by week in the study. Sixty-seven symptomatic treatment failures cases were successfully analysed by both MSP2 and pfPK2. The mean number of clones at enrolment and day of failure is constant throughout the 4 study weeks. At all times points there were multiple clones recrudescing (treatment failures) in individual subjects. The mean number of clones recrudescing was greatest in the first week of the study and declined over time but remained greater than 1 for the whole period. There were 5 examples where patients were found to have between 5–6 identical pfPK2 alleles plus 2–3 MSP2 alleles which matched on enrolment and day of failure, all occurred within the first week of treatment.

**Figure 4:**
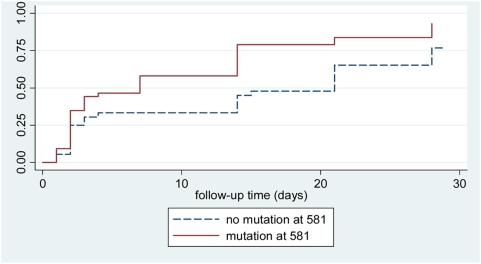
Cumulative proportions of treatment failure of Sulphadoxine-Pyrimethamine in symptomatic 6–59 month old children with malaria parasites carrying or not-carrying the A581G mutation in the *dhps* gene at enrolment. Unadjusted effect *dhps* gene mutation at 581, p-value = 0.012.

The prevalence of mutations at each codon are shown in table 3 for *dhfr* and table 4 for *dhps* in both groups and prevalence of haplotypes in table 5 for *dhfr* and *dhps*. There were no mutations found at codon 164 in the *dhfr* gene but 54% of parasites in the symptomatic group carried the 581 G mutation in the *dhps* gene on enrollment day. The ratio of 581 G mutation to 581 A wild type increased from enrollment day to day of failure (0.78 and 0.93 respectively) but this change was not statistically significant (p = 0,5). Direct sequencing confirmed the presence of mutations at codons 436, 437, 540 and 581 and did not reveal any additional mutations. In the symptomatic group 96% of parasites carried the triple *dhfr* mutant haplotype (mutants at codons 51, 59 and 108) (Table 4) showing near saturation of this haplotype. As we would be unlikely to demonstrate any further selection in *dhfr* haplotype we did not analyse day of failure samples for these mutations in the symptomatic study. Parasites with the double mutations in the *dhps* gene at codon positions 437 and 540 were found in 65% and triple mutations with the additional mutation at position 581 were found in 55% of samples collected at enrolment. Molecular data for the asymptomatic study were too few to analyze (6/25 samples amplified for the day of enrolment) and were excluded from further analysis. From samples from the symptomatic study a significant trend for increased and earlier failure was seen with those carrying the 581G mutation prior to treatment (Figure 4). However, in a regression model, factors associated with failure in the symptomatic group were presence of three mutations in the *dhfr* gene, age <2 years and high parasite density (Table 6) and not the presence of the 581 G mutations. The results of the model did not change when looking at treatment failure on or before day 3 versus failure after day 3 or treatment failure on or before day 14 versus failure after day 14 (data not shown).

**Table 3:** Prevalence of mutations in *dhfr* gene at day of enrolment and day of failure in all study children.

	Enrolment day symptomatic cases				Day of failure symptomatic cases			
	N = 84							
*codon*	**51**	**59**	**108**	**164**	**51**	**59**	**108**	**164**
*sensitive*	**N**	**C**	**S**	**I**	Not done	Not done	Not done	Not done
	9 (10.7%)	11 (13.1%)	3 (3.6%)	84 (100%)				
*mutant*	**I**	**R**	**N**	**L**	Not done	Not done	Not done	Not done
	81 (96.4%)	79 (94.0%)	82 (97.6%)	0 (0%)				

	Enrolment day asymptomatic cases				Day of failure asymptomatic cases			
	N = 6				N = 6			
*codon*	**51**	**59**	**108**	**164**	**51**	**59**	**108**	**164**
*sensitive*	**N**	**C**	**S**	Not done	**N**	**C**	**S**	Not done
	4 (66.7%)	2 (33.3%)	2 (33.3%)		0 (0%)	0 (0%)	0 (0%)	
*mutant*	**I**	**R**	**N**	Not done	**I**	**R**	**N**	Not done
	5 (83.3%)	6 (100%)	6 (100%)		6 (100%)	6 (100%)	6 (100%)	

**Table 4:** Prevalence of mutations in *dhps* gene at day of enrolment and day of failure in all study children.

	Enrolment day symptomatic cases					Day of failure symptomatic cases				
	N = 87					N = 67				
*codon*	**436**	**437**	**540**	**581**	**613**	**436**	**437**	**540**	**581**	**613**
*sensitive*	**S**	**A**	**K**	**A**	**A**	**S**	**A**	**K**	**A**	**A**
	85 (97.7%)	13 (14.9%)	14 (16.1%)	60 (69.0%)	87 (100%)	67 (100%)	3 (4.5%)	3 (4.5%)	44 (65.7%)	67 (100%)
*mutant*	**A,C,F**	**G**	**E**	**G**	**S,T**	**A,C,F**	**G**	**E**	**G**	**S,T**
	8 (9.2%)	83 (95.4%)	82 (94.3%)	47 (54.0%)	0 (0%)	1 (1.5%)	67 (100%)	67 (100%)	41 (61.2%)	0 (0%)

	Enrolment day asymptomatic cases					Day of failure asymptomatic cases				
	N = 9					N = 11				
*codon*	**436**	**437**	**540**	**581**	**613**	**436**	**437**	**540**	**581**	**613**
*sensitive*	**S**	**A**	**K**	**A**	**A**	**S**	**A**	**K**	**A**	**A**
	9 (100%)	0 (0%)	1 (11.1%)	7 (77.8%)	9 (100%)	11 (100%)	0 (0%)	0 (0%)	8 (72.7%)	11 (100%)
*mutant*	**A,C,F**	**G**	**E**	**G**	**S,T**	**A,C,F**	**G**	**E**	**G**	**S,T**
	0 (0%)	9 (100%)	9 (100%)	4 (44.4%)	0 (0%)	0 (0%)	11 (100%)	11 (100%)	6 (54.5%)	0 (0%)

**Table 5:** Prevalence of point mutation haplotypes in *dhfr* and *dhps* at day of enrolment in symptomatic and asymptomatic children.

Number of samples	N = 84*
***dhfr*** ** mutations**	
Sensitive (NCS)	3 (3.6%)
Single (NCN)	0 (0%)
Double (ICN)	8 (9.5%)
Double (NRN)	1 (1.2%)
Triple (IRN)	81 (96.4%)
Mixed infections (unable to determine haplotype)	0 (0%)

Number of samples	N = 87*
***dhps*** ** mutations**	
Sensitive (SAKA)	4 (4.6%)
Single (436A or C only = AAKA or CAKA)	8 (9.2%)
Single (437G only = SGKA)	0 (0%)
Single (540E only = SAEA)	0 (0%)
Single (581G only = SAKG)	1 (1.1%)
Double (437G+540E only = SGEA)	55 (63.2%)
Triple (437G+540E+581G = SGEG)	48 (55.2%)
Mixed infections (unable to determine haplotype)	2 (2.3%)

* includes mixed infections when haplotypes could be ascertained (9 for *dhfr* and 31 for *dhps*).

**Table 6:** Association between mutations and treatment failure.

Parameter	Category	Total (n)	All failures (%)	Crude OR	Adjusted OR^*^	P-value
*dhfr* mutation	No/single/double	6	3	1.0	1.0	
	Triple	72	66	11.0 (1.8–66.9)	12.0 (1.1–128)	0.040
*dhps A581G* mutation	No mutation	36	28	1.0	1.0	
	Mutation	43	40	3.8 (0.9–15.6)	4.9 (0.7–34.8)	0.116
Age	<2 yrs	29	27	1.0	1.0	
	2–4 yrs	56	45	0.4 (0.1–1.8)	0.04 (0.002–0.9)	0.046
Parasite density (Asexual forms/ul)	<28000	29	21	1.0	1.0	
	(28000–67000)	27	26	9.9(1.1–85.6)	20.5(1.2–330)	0.033
	>68000	7	25	4.8(0.9–4.9)	10.4(1.1–95.8)	0.039

Adjusted odds ratio: logistic regression model including age, parasite density and mutations of DHFR and DHPS. Asymptomatic cases not included in model due to too few cases with complete molecular results (6/25).

### Distribution of the *dhps* position 581 mutation in Africa

We identified 107 surveys in 59 unique geographical localities in Africa, where *P. falciparum* isolates have been tested for the 581G mutation. The 41 references which describe them are listed in supplementary online material (References S1). In addition to this list a map with embedded survey details and links to the original references is available online at www.drugresistancemaps.org.

**Figure 5:**
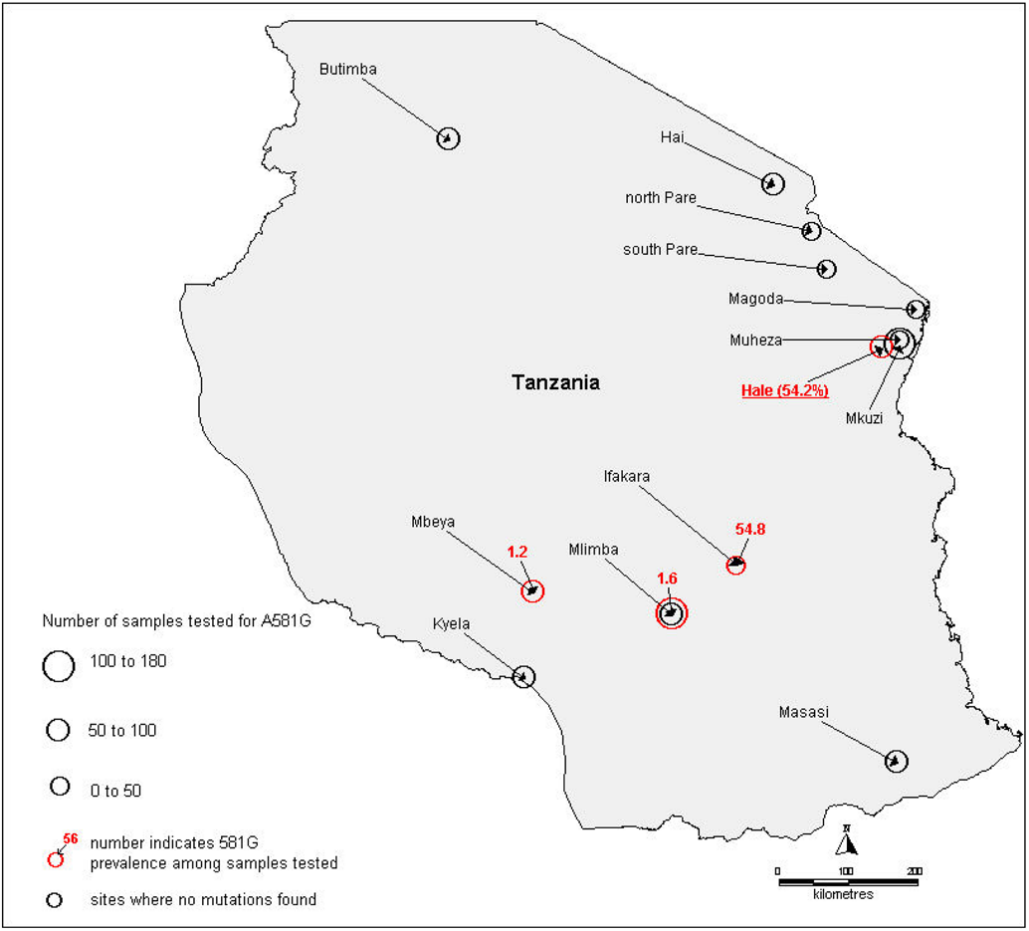
Distribution of 581G mutation in Tanzania.

**Figure 6:**
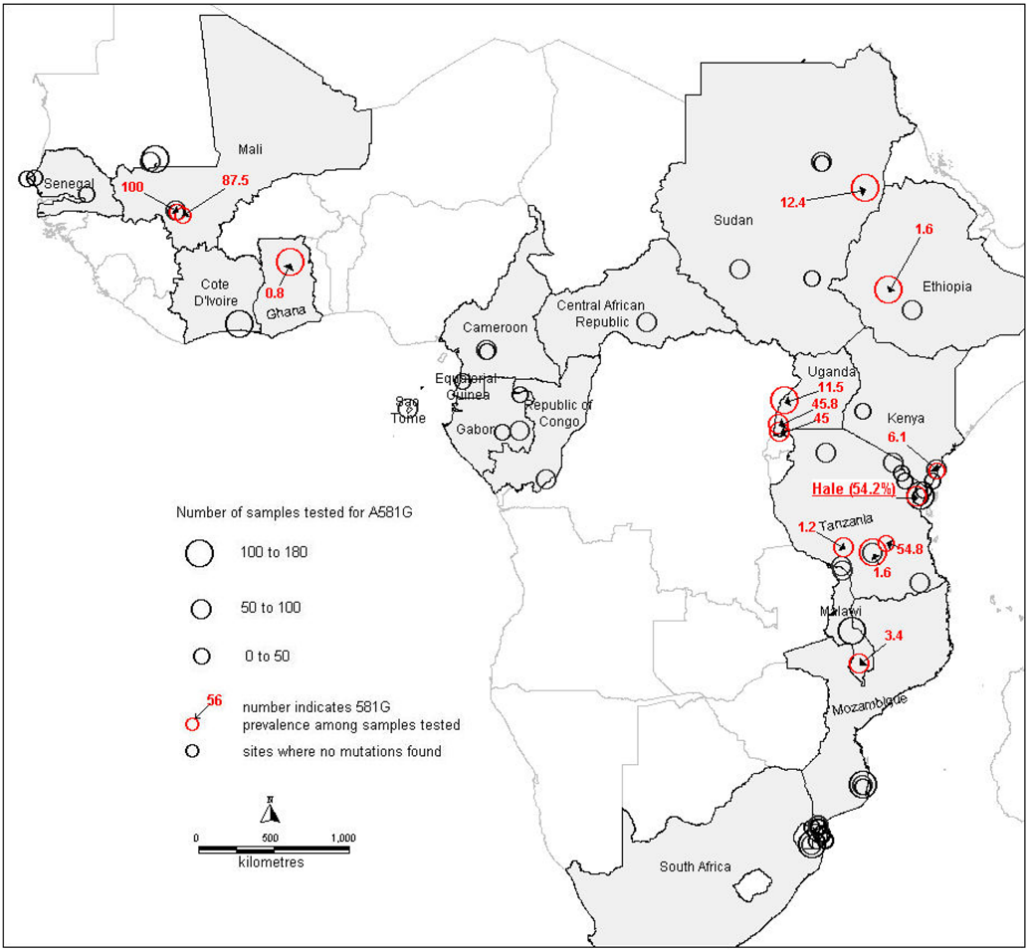
Distribution of 581G mutation in Africa.

The occurrence of the 581G mutation is comparatively rare in both Tanzania (figure 5) and in Africa as a whole (figure 6). Of 4932 isolates tested in total, 96.5% carried the 581 A wild type genotype. Survey sites where 581G mutations *were not* found are identified in the maps as black circles whereas those in which 581G was found are shown as red circles with the prevalence of the mutation indicated.

In Tanzania, the 581G mutation was observed in just 3 of 16 previous surveys. Our study in Hale found 581G in 54% of isolates (n = 84). In Ifakara[31] in 1997, 27.8% of isolates (n = 18) were found to carry the mutant while Mlimba[32] and Mbeya[33] had low prevalence of 1.6% (n = 128) and 1.2% (n = 81) respectively. Surveys in Butimba (n = 57), Mlimba (n = 59), Kyela (n = 67), Masasi (n = 73), Mkuzi (n = 127), reported no 581G mutations [8], and similarly a survey in Muheza (n = 28) [34].

In Africa as a whole the A581G mutation has been observed in only 14 of a total of 106 surveys. The surveys that had observed A581G mutation span nine countries namely Ethiopia[35] 1.6% (n = 124), Ghana[36] 0.8% (n = 126), Kenya[31] 6.1% (n = 33), Malawi[37] 3.4% (n = 89), Mali[31] 100% (n = 10) and 87% (n = 8), Sudan [38]12.4% (n = 153), Tanzania (see above) and Uganda[24], [39], [40] 11.5% (n = 122) and 45.8% (n = 72) 45% (n = 60).

## Discussion

The results of this study demonstrate clearly that use of SP for the treatment of *P falciparum* malaria is dangerous in the area where our study was done. SP had no effect on the course of illness in four cases, three in the symptomatic group and one in the asymptomatic group, who developed severe disease within 3 days of post treatment. The efficacy of SP resulted in similarly poor parasitologic clearance in 2–10 month old asymptomatic infants when compared with 6–59 month old symptomatic children. The WHO recommends changing first line treatment when day 28 failure rates exceed 10%[9]. In this study, early treatment failures of symptomatic children were 38.8% and by day 28 a staggering 82% required rescue treatment. Shortly after the study was stopped artemether-lumefantrine (ALU) became the first-line treatment for uncomplicated malaria in Tanzania, although SP remains widely available through private drug stores and is frequently used.

The primary purpose of this study was to determine if SP was more efficacious in asymptomatic parasitaemic infants than in symptomatic children even though the drug was failing as a treatment for the latter group. This has been shown to be the case for pregnant women in areas of moderate SP resistance where SP may still be effective for treatment or prevention even though it fails when used for the treatment of symptomatic children[41], [42]. Because the study had to be terminated prematurely on the grounds of the unexpectedly high failure rates in symptomatic children and asymptomatic infants, it was underpowered to show a difference between the two study groups. Estimates of efficacy had confidence intervals of 10% and 20% in the symptomatic and asymptomatic study groups respectively and the study was powered to detect only a 30% or more difference between groups. Nevertheless, the findings suggest that there was no major difference in response to SP between symptomatic older children and asymptomatic infants contrasting with the major differences observed in comparisons between asymptomatic pregnant women and symptomatic children. This difference may be due to the fact that infants in the second half of the first year of life, in contrast to pregnant women, have less naturally acquired immunity to malaria. In pregnant women, but not infants, naturally acquired immunity may be sufficient to clear low density infections when parasites are exposed to an only partially effective drug. Evidence from high transmission sites suggests that maternal protective factors do protect infants against recrudescence[43], [44], however, how these protective factors compare to maternal immunity itself is not known. Although the study split children into symptomatic and asymptomatic groups the difference between these groups may only be that the asymptomatic children were at an earlier stage of the disease. In children in a moderate transmission setting in Uganda approximately 50% of asymptomatic parasitaemic children progressed to clinical disease within 30 days of detection[45]. This group of children, however, is relevant for this study as they represent the population who would be treated with IPTi. This study examined the treatment effect of SP and did not address the ability for SP to prevent blood stage infection, i.e. the prophylactic effect[10]. Whether SP has any efficacy in the study area when used for IPTi (both treatment and prophylactic effects[10]) will be determined in an ongoing IPTi study that will be reported in early 2009 (clintrial.gov identifier: NCT00158574).

Within 8 years the level of SP resistance in the study area has increased at an alarming rate. The day 28 ACPR has decreased from 55% in 1999 [34] to the current situation of 18%. This change is likely to have been driven, at least in part, by the use of SP as first line treatment for malaria from 2001 to 2007. Now that first line treatment for malaria has changed to combination therapy across most of sub- Saharan Africa, the selection pressure for resistance to SP will have been reduced. Nevertheless the rapid increase in SP resistance is a concern and an alternative drug needs to be developed for all SP-based prevention programs.

The molecular work was limited by the number of clones in the samples and the sensitivity of the methods to detect masked clones. In the symptomatic group 4 new infections were detected in the first 3 days post-treatment. This is a highly unlikely result and is probably due to masked clones resistant to SP becoming dominant post treatment, a common criticism of PCR-correction. This problem was also likely to interfere with the interpretation of new infections when comparing the symptomatic and asymptomatic groups as the symptomatic group was more likely to have heavier parasite densities and more clones. Thus, in the symptomatic group a higher proportion of recrudescences would be wrongly called new infections. The number of clones detected in samples was high and in many cases these represented multiple clones recrudescing reflecting the high transmission and high prevalence of resistance genotypes circulating in the study area.

Further *in vivo* efficacy studies of SP are unjustifiable in areas where the level of SP resistance is known to be high. In such situations, studies of molecular markers of resistance play a key role in measuring patterns of resistance as there is strong evidence that mutations in *dhfr* and *dhps* genes are associated with *P. falciparum* resistance to SP. Previous reports from Africa have demonstrated an association between moderate levels of resistance to SP and triple mutation in the *dhfr* [16], [17], [18] and double mutations in the *dhps* genes[18] and this association was found in our study. In South East Asia the presence of high level resistance to antifolates has been associated with the presence on an additional mutation at position 164 on the *dhfr* gene[20]. In the face of the very high level of SP resistance found in Hale we considered it possible that parasites carrying this mutation had emerged in north-eastern Tanzania. However, this did not turn out to be the case and no parasites carrying the 164 mutation were found. Instead 55% of haplotypes, when they could be ascertained, in addition to the 437 and 540 mutations had a mutation at codon 581 in the parasite *dhps* gene and there was some evidence that the presence of this mutation in parasites at the start of treatment was associated earlier treatment failure. Biological plausibility of this is backed by the findings that the 581G single mutant offers resistance in *vitro* and that similar mutations in bacteria produce resistance to sulfa drugs[14]. The *dhps* triple is recognized in South America, where it is associated with SP treatment failure[46]. Parasites carrying the 581G mutation are widely distributed in Tanzania (figure 5) and elsewhere in Africa (figure 6). The evidence of prevalence of this mutation is patchy with few studies looking at this locus and those that have show considerable differences in prevalence between countries. It is likely that the large drug pressure caused when SP was the first line drug for uncomplicated malaria that has driven the rise in frequency of first the *dhps* double and now the more resistant *dhps* triple mutation. This early failure may prove to be pertinent in the case of IPTp. ter Kuile and colleagues[41] found that SP IPTp had reasonable protective efficacy against low birth-weight even in areas where the day 14 treatment failure rate of SP in symptomatic children was as high as 35%. However, they observed that the duration of prophylaxis of SP had reduced from 4 to 2 weeks. In areas where the 581 mutation is present in conjunction with the triple *dhfr* and double *dhps* mutants, it is possible that the duration of prophylaxis given by SP will be further reduced and this may affect the protective efficacy of SP preventative strategies. Further work is needed to determine the geographical spread of this mutant and if the presence of this mutation in combination with the *dhfr* triple and *dhps* double confers increased and earlier failure of cases treated with SP.

## Supporting Information

References S1 References collected by systematic review to produce maps of the prevalence of the 581 dhps Mutation in figures 5 and 6
(0.04 MB DOC)

Checklist S1 CONSORT Checklist
(0.06 MB DOC)

Protocol S1 These are the 2 study protocols which were approved by the IRBs
(0.01 MB ZIP)
