# Supplementary material for: High Resistance of Plasmodium falciparum to Sulphadoxine/Pyrimethamine in Northern Tanzania and the Emergence of dhps Resistance Mutation at Codon 581
Source: PLoS One. 2009 Feb 24;4(2):e4569. doi: 10.1371/journal.pone.0004569 (PMC2644264; doi:10.1371/journal.pone.0004569)
Supplement: References S1 — References collected by systematic review to produce maps of the prevalence of the 581 dhps Mutation in figures 5 and 6 (0.04 MB DOC) [file pone.0004569.s001.doc]

**Supplementary Information: Gesase *et al*., 2008**

1. A-Elbasit IE, Alifrangis M, Khalil IF, *et al*. The implication of dihydrofolate reductase and dihydropteroate synthetase gene mutations in modification of *Plasmodium falciparum* characteristics. Malar J 2007;6:108

2. Alker AP, Mwapasa V, Purfield A, *et al*. Mutations associated with sulfadoxine-pyrimethamine and chlorproguanil resistance in *Plasmodium falciparum* isolates from Blantyre, Malawi. Antimicrob Agents Chemother 2005;49:3919-21

3. Anderson TJ, Nair S, Jacobzone C, Zavai A and Balkan S. Molecular assessment of drug resistance in *Plasmodium falciparum* from Bahr El Gazal province, Sudan. Trop Med Int Health 2003;8:1068-73

4. Aubouy A, Jafari S, Huart V, *et al*. DHFR and DHPS genotypes of *Plasmodium falciparum* isolates from Gabon correlate with in vitro activity of pyrimethamine and cycloguanil, but not with sulfadoxine-pyrimethamine treatment efficacy. J Antimicrob Chemother 2003;52:43-9

5. Basco LK, Tahar R and Ringwald P. Molecular basis of in vivo resistance to sulfadoxine-pyrimethamine in African adult patients infected with *Plasmodium falciparum* malaria parasites. Antimicrob Agents Chemother 1998a;42:1811-4

6. Basco LK, Ringwald P. Molecular epidemiology of malaria in Yaounde, Cameroon II. Baseline frequency of point mutations in the dihydropteroate synthase gene of Plasmodium falciparum. Am J Trop Med Hyg 1998b;58:374-7

7. Basco LK, Tahar R, Keundjian A and Ringwald P. Sequence variations in the genes encoding dihydropteroate synthase and dihydrofolate reductase and clinical response to sulfadoxine-pyrimethamine in patients with acute uncomplicated falciparum malaria. J Infect Dis 2000;182:624-8

8. Berzosa PJ, Puente S and Benito A. Malaria cure with sulphadoxine/pyrimethamine combination in 12 semi-immune adults from West-Central Africa with high rates of point mutations in *Plasmodium falciparum* dhfr and dhps genes. Parasitol Res 2005;97:287-9

9. Bwijo B, Kaneko A, Takechi M, *et al*. High prevalence of quintuple mutant dhps/dhfr genes in *Plasmodium falciparum* infections seven years after introduction of sulfadoxine and pyrimethamine as first line treatment in Malawi. Acta Trop 2003;85:363-73

10. Cravo. High frequency of the genetic polymorphisms associated with sulfadoxine-pyrimethamine resistance, among *Plasmodium falciparum* isolates from Sao Tome and Principe, West Africa, 2004

11. Curtis J, Duraisingh MT and Warhurst DC. In vivo selection for a specific genotype of dihydropteroate synthetase of *Plasmodium falciparum* by pyrimethamine-sulfadoxine but not chlorproguanil-dapsone treatment. J Infect Dis 1998;177:1429-33

12. Diourte Y, Djimde A, Doumbo OK, *et al*. Pyrimethamine-sulfadoxine efficacy and selection for mutations in *Plasmodium falciparum* dihydrofolate reductase and dihydropteroate synthase in Mali. Am J Trop Med Hyg 1999;60:475-8

13. Djaman JA, Mazabraud A and Basco L. Sulfadoxine-pyrimethamine susceptibilities and analysis of the dihydrofolate reductase and dihydropteroate synthase of *Plasmodium falciparum* isolates from Cote d'Ivoire. Ann Trop Med Parasitol 2007;101:103-12

14. Eberl KJ, Jelinek T, Aida AO, *et al*. Prevalence of polymorphisms in the dihydrofolate reductase and dihydropteroate synthetase genes of *Plasmodium falciparum* isolates from southern Mauritania. Trop Med Int Health 2001;6:756-60

15. Gebru-Woldearegai T, Hailu A, Grobusch MP and Kun JF. Molecular surveillance of mutations in dihydrofolate reductase and dihydropteroate synthase genes of *Plasmodium falciparum* in Ethiopia. Am J Trop Med Hyg 2005;73:1131-4

16. Jelinek T, Ronn AM, Lemnge MM, *et al*. Polymorphisms in the dihydrofolate reductase (DHFR) and dihydropteroate synthetase (DHPS) genes of *Plasmodium falciparum* and in vivo resistance to sulphadoxine/pyrimethamine in isolates from Tanzania. Trop Med Int Health 1998;3:605-9

17. Jelinek T, Kilian AH, Kabagambe G and von Sonnenburg F. *Plasmodium falciparum* resistance to sulfadoxine/pyrimethamine in Uganda: correlation with polymorphisms in the dihydrofolate reductase and dihydropteroate synthetase genes. Am J Trop Med Hyg 1999a;61:463-6

18. Jelinek T, Kilian AH, Westermeier A, *et al*. Population structure of recrudescent *Plasmodium falciparum* isolates from western Uganda. Trop Med Int Health 1999b;4:476-80

19. Khalil I, Alifrangis M, Ronn AM, *et al*. Pyrimethamine/sulfadoxine combination in the treatment of uncomplicated falciparum malaria: relation between dihydropteroate synthase/dihydrofolate reductase genotypes, sulfadoxine plasma levels, and treatment outcome. Am J Trop Med Hyg 2002;67:225-9

20. Khalil I, Ronn AM, Alifrangis M, Gabar HA, Satti GM and Bygbjerg IC. Dihydrofolate reductase and dihydropteroate synthase genotypes associated with in vitro resistance of *Plasmodium falciparum* to pyrimethamine, trimethoprim, sulfadoxine, and sulfamethoxazole. Am J Trop Med Hyg 2003;68:586-9

21. Kun JF, Lehman LG, Lell B, Schmidt-Ott R and Kremsner PG. Low-dose treatment with sulfadoxine-pyrimethamine combinations selects for drug-resistant *Plasmodium falciparum* strains. Antimicrob Agents Chemother 1999;43:2205-8

22. Lynch C, Pearce R, Pota H, *et al*. Emergence of highly resistant *dhfr* alleles in *P. falciparum* populations of SW Uganda. Journal of Infectious Diseases 2008: in press.

23. Mawili-Mboumba DP, Ekala MT, Lekoulou F and Ntoumi F. Molecular analysis of DHFR and DHPS genes in *P. falciparum* clinical isolates from the Haut--Ogooue region in Gabon. Acta Trop 2001;78:231-40

24. Mbugi EV, Mutayoba BM, Malisa AL, Balthazary ST, Nyambo TB and Mshinda H. Drug resistance to sulphadoxine-pyrimethamine in *Plasmodium falciparum* malaria in Mlimba, Tanzania. Malar J 2006;5:94

25. Menard D, Djalle D, Yapou F, Manirakiza A and Talarmin A. Frequency distribution of antimalarial drug-resistant alleles among isolates of *Plasmodium falciparum* in Bangui, Central African Republic. Am J Trop Med Hyg 2006;74:205-10

26. Mockenhaupt FP, Teun Bousema J, Eggelte TA, *et al*. *Plasmodium falciparum* dhfr but not dhps mutations associated with sulphadoxine-pyrimethamine treatment failure and gametocyte carriage in northern Ghana. Trop Med Int Health 2005;10:901-8

27. Mugittu K, Ndejembi M, Malisa A, *et al*. Therapeutic efficacy of sulfadoxine-pyrimethamine and prevalence of resistance markers in Tanzania prior to revision of malaria treatment policy: *Plasmodium falciparum* dihydrofolate reductase and dihydropteroate synthase mutations in monitoring in vivo resistance. Am J Trop Med Hyg 2004;71:696-702

28. Mutabingwa T, Nzila A, Mberu E, *et al*. Chlorproguanil-dapsone for treatment of drug-resistant falciparum malaria in Tanzania. Lancet 2001;358:1218-23

29. Ndiaye D, Daily JP, Sarr O, *et al*. Mutations in *Plasmodium falciparum* dihydrofolate reductase and dihydropteroate synthase genes in Senegal. Trop Med Int Health 2005;10:1176-9

30. Ndounga M, Tahar R, Basco LK, Casimiro PN, Malonga DA and Ntoumi F. Therapeutic efficacy of sulfadoxine-pyrimethamine and the prevalence of molecular markers of resistance in under 5-year olds in Brazzaville, Congo. Trop Med Int Health 2007;12:1164-1171

31. Nzila AM, Mberu EK, Sulo J, *et al*. Towards an understanding of the mechanism of pyrimethamine-sulfadoxine resistance in Plasmodium falciparum: genotyping of dihydrofolate reductase and dihydropteroate synthase of Kenyan parasites. Antimicrob Agents Chemother 2000a;44:991-6

32. Nzila AM, Nduati E, Mberu EK, *et al*. Molecular evidence of greater selective pressure for drug resistance exerted by the long-acting antifolate Pyrimethamine/Sulfadoxine compared with the shorter-acting chlorproguanil/dapsone on Kenyan Plasmodium falciparum. J Infect Dis 2000b;181:2023-8

33. Omar SA, Adagu IS and Warhurst DC. Can pretreatment screening for dhps and dhfr point mutations in *Plasmodium falciparum* infections be used to predict sulfadoxine-pyrimethamine treatment failure? Trans R Soc Trop Med Hyg 2001;95:315-9

34. Pearce RJ, Drakeley C, Chandramohan D, Mosha F and Roper C. Molecular determination of point mutation haplotypes in the dihydrofolate reductase and dihydropteroate synthase of *Plasmodium falciparum* in three districts of northern Tanzania. Antimicrob Agents Chemother 2003;47:1347-54

35. Plowe CV, Cortese JF, Djimde A, *et al*. Mutations in *Plasmodium falciparum* dihydrofolate reductase and dihydropteroate synthase and epidemiologic patterns of pyrimethamine-sulfadoxine use and resistance. J Infect Dis 1997;176:1590-6

36. Raman J, Sharp B, Kleinschmidt I, *et al*. Differential Effect of Regional Drug Pressure on Dihydrofolate Reductase and Dihydropteroate Synthetase Mutations in Southern Mozambique. Am J Trop Med Hyg 2008;78:256-261

37. Roper C, Pearce R, Bredenkamp B, *et al*. Antifolate antimalarial resistance in southeast Africa: a population-based analysis. Lancet 2003;361:1174-81

38. Schönfeld M, Barreto Miranda I, Schunk M, *et al*. Molecular surveillance of drug-resistance associated mutations of *Plasmodium falciparum* in south-west Tanzania. Malar J 2007;6:2

39. Schunk M, Kumma WP, Miranda IB, *et al*. High prevalence of drug-resistance mutations in *Plasmodium falciparum* and Plasmodium vivax in southern Ethiopia. Malar J 2006;5:54

40. van den Broek IV, Gatkoi T, Lowoko B, Nzila A, Ochong E and Keus K. Chloroquine, sulfadoxine-pyrimethamine and amodiaquine efficacy for the treatment of uncomplicated *Plasmodium falciparum* malaria in Upper Nile, south Sudan. Trans R Soc Trop Med Hyg 2003;97:229-35

41. Wang P, Lee CS, Bayoumi R, *et al*. Resistance to antifolates in *Plasmodium falciparum* monitored by sequence analysis of dihydropteroate synthetase and dihydrofolate reductase alleles in a large number of field samples of diverse origins. Mol Biochem Parasitol 1997;89:161-77
