# Supplementary material for: High Resistance of Plasmodium falciparum to Sulphadoxine/Pyrimethamine in Northern Tanzania and the Emergence of dhps Resistance Mutation at Codon 581
Source: PLoS One. 2009 Feb 24;4(2):e4569. doi: 10.1371/journal.pone.0004569 (PMC2644264; doi:10.1371/journal.pone.0004569)
Supplement: Protocol S1 — These are the 2 study protocols which were approved by the IRBs (0.01 MB ZIP) [file pone.0004569.s003.zip › Protocols/Protocol 2-10 month study ammended 23 feb 06.doc]

**Protocol for *in vivo* drug efficacy study of sulfadoxine/pyrimethamine in asymptomatic 2-10 months of age children with parasitaemia, in Kilimanjaro IPTi study area using modified WHO methodology**

Version 2 Amended 28^th^ January 2006. Changes are highlighted.

Children 2-10 months of age who voluntarily attend the Maternal Child Health (MCH) for immunization or weighing will be offered screening using an informed consent procedure. The parents of those children who meet the inclusion criteria during the screening procedure (see section below) will be offered enrolment into the study. If the parent/guardian expresses interest in enrolment, a full explanation of the study protocol will be provided to the parent/guardian in the local language as part of the second informed consent procedure. Consent forms approved by Institutional Review Board (IRB) of the institution(s) responsible for the conduct of the IPTi and *in vivo* trials will be presented for witnessed written consent and those unable to write will be asked to provide a witnessed thumb print by the patient or guardian. The study physician will then examine the patient and fill out the Case Record Form (CRF).

**Inclusion criteria.**

- Aged 2-10 months
- Weight of 4.5 Kgs or greater.
- Not enrolled in IPTi trial
- Absence of severe malnutrition (defined as a child whose weight-for-height is below 3 standard deviations of less than 70% of the median of WHO reference values, or who has symmetrical edema involving at least the feet)
- A slide-confirmed infection with *P. falciparum* only (i.e. no mixed infections)
- Any parasite density assessed by research microscopists.
- Absence of general danger signs among children < 5 years (inability to drink or breastfeed; vomiting everything; recent history of convulsions; lethargy or unconsciousness; inability to sit or stand up) or other signs of severe and complicated falciparum malaria according to WHO definitions
- Measured axillary temperature < 37.5 °C
- Ability to attend stipulated follow-up visits
- Informed consent provided by parent/guardian
- Absence of history of hypersensitivity reactions to SP.

Exclusion criteria are not listed separately in the WHO protocol, as they are the opposite of the inclusion criteria. However, for the sake of clarity, these criteria are listed below:

**Exclusion criteria**

- Aged < 2 or >10 months
- Weight less than 4.5 Kgs.
- Enrolled in IPTi trial
- Severe malnutrition (defined as a child whose weight-for-height is below 3 standard deviations of less than 70% of the median of WHO reference values, or who has symmetrical edema involving at least the feet)
- No slide confirmed infection with *P. falciparum* or a mixed infection that includes a non-*P. falciparum* species
- Presence of general danger signs among children (inability to drink or breastfeed; vomiting everything; recent history of convulsions; lethargy or unconsciousness; inability to sit or stand up) or other signs of severe and complicated falciparum malaria according to WHO definitions
- Measured axillary temperature > 37.5 °C
- Inability to attend stipulated follow-up visits
- Unwilling to provide informed consent provided by parent/guardian
- History of hypersensitivity reactions to SP.

Physician investigators will examine these patients to ascertain that they fit the above criteria. Patients with any of the above exclusion characteristics or who are for any other reason determined to be too ill for inclusion in the study, or who decline to participate in the study, will receive standard and appropriate treatment, according to MOH guidelines.

The trial will be conducted in an open-label (unblinded) fashion. All subjects will be observed for at least half an hour to monitor for adverse reactions and to make sure that the medicine is not vomited. If vomiting occurs within 30 min, a full dose will be re-administered and if vomiting occurs between 30 min and 1 hour, half the dose will be re-administered. If a child vomits a second time they will be excluded from the study and given rescue treatment (see IPTi Efficacy study- study drug doses and rescue treatment). All drug doses will be observed. Children with vomiting who appear dehydrated will be re-hydrated with oral rehydration solution or intravenous fluids depending on the judgment of the clinical staff.

**History and physical data**. Symptoms, prior anti-malarial drug use and physical examination data will be recorded on standard data forms– also known as a Case Record Form or CRF.

**Sample collection*.*** Subjects will have a single finger prick. Samples will be collected for 1) Rapid diagnostic test for malaria, 2)Malaria blood films (one thick film and one thin film), 3) blood-onto-filter paper samples and 4) haemoglobin estimation using a Hemocue at enrolment. Blood films will be Giemsa-stained and read for initial parasitemia screening, parasite speciation, and quantification. Filter papers will be air-dried and stored for analysis of *P. falciparum* genetic markers for drug resistance.

**Follow up*.*** With enrollment occurring on day 0, subjects will be followed actively on days 1,2,3 7, 14, 21, and 28 by invitation to report to the clinic, and passively by 24-hour availability of a study clinician to evaluate and treat study subjects. At days 7, 14 and 28 and passive follow-up times malaria blood films and filter paper strips will be obtained and brief history and physical evaluations made to seek signs or symptoms of persistent or recrudescent malaria. If the study child has a fever or history of fever on follow up days 1, 2, 3 or 21 they will also have a blood samples taken for a blood slide and filter paper. Filter papers will be used to look for parasite (*P. falciparum*) mutations linked to drug resistance and to examine parasite genotype with the aim of distinguishing recrudescence from re-infection.

A study clinician will promptly evaluate cases of post- treatment appearance of malaria symptoms and parasitaemia. If a child is sick and remains parasitaemic they will be treated with the appropriate antimalarial (Coartem). Cases of severe malaria in any subject evaluated at any time, including the first three days after treatment and whether or not they are enrolled in the study, will be fully evaluated and admitted to hospital for appropriate medical care.

**Data analysis and interpretation**

Treatment outcome will be categorized as adequate parasitological response (APR) at day 28 corrected for recrudescence and re-infection using PCR. We will examine APR at day 7 and 14 for descriptive purposes.

Adequate Parasitological Response (APR) will be defined as:

1. Absence of parasites from peripheral blood thick film after examining 100 high power fields.

All day 0 samples from cases meeting criteria for resistance or treatment failure will be analyzed for molecular markers of antimalarial drug resistance as will the sample from the day that resistance was identified. In addition, two baseline sensitive/adequate clinical response samples will be randomly chosen as unmatched controls for each case of resistance or treatment failure in order to compare the prevalence of these molecular markers.
