# Supplementary material for: High Resistance of Plasmodium falciparum to Sulphadoxine/Pyrimethamine in Northern Tanzania and the Emergence of dhps Resistance Mutation at Codon 581
Source: PLoS One. 2009 Feb 24;4(2):e4569. doi: 10.1371/journal.pone.0004569 (PMC2644264; doi:10.1371/journal.pone.0004569)
Supplement: Protocol S1 — These are the 2 study protocols which were approved by the IRBs (0.01 MB ZIP) [file pone.0004569.s003.zip › Protocols/Protocol 6-59 month study ammended no MQ 20 June 06.doc]

**Protocol for 6-59 months of age *in vivo* drug efficacy study of sulfadoxine/pyrimethamine, and chlorporguanil/dapsone in Kilimanjaro IPTi study area using standard WHO methodology**

Version 3 Amended 20^th^ June 2006. Changes are highlighted.

Children 6-59 months of age suffering from all acute illnesses who voluntarily present to the clinic will be examined by study physicians. The parent/guardian of those children having symptoms consistent with uncomplicated malaria will be offered screening using an informed consent procedure. The parents of those children who meet the inclusion criteria during the screening procedure (see section below) will be offered enrolment into the study. If the parent/guardian expresses interest in enrolment, a full explanation of the study protocol will be provided to the parent/guardian in the local language as part of the second informed consent procedure. Consent forms approved by Institutional Review Board (IRB) of the institution(s) responsible for the conduct of the IPTi and *in vivo* trials will be presented for witnessed written consent and those unable to write will be asked to provide a witnessed thumb print by the patient or guardian. The study physician will then examine the patient and fill out the Case Record Form (CRF).

**Inclusion criteria.**

- Aged 6-59 months
- Weight of 4.5 Kgs or greater.
- Not enrolled in IPTi trial
- Absence of severe malnutrition (defined as a child whose weight-for-height is below 3 standard deviations of less than 70% of the median of WHO reference values, or who has symmetrical edema involving at least the feet)
- A slide-confirmed infection with *P. falciparum* only (i.e. no mixed infections)
- Initial parasite density between 2,000 and 200,000 asexual parasites per microliter.
- Absence of general danger signs among children < 5 years (inability to drink or breastfeed; vomiting everything; recent history of convulsions; lethargy or unconsciousness; inability to sit or stand up) or other signs of severe and complicated falciparum malaria according to WHO definitions
- Measured axillary temperature ≥37.5 °C
- Ability to attend stipulated follow-up visits
- Informed consent provided by parent/guardian
- Absence of history of hypersensitivity reactions to any of the drugs being evaluated

Exclusion criteria are not listed separately in the WHO protocol, as they are the opposite of the inclusion criteria. However, for the sake of clarity, these criteria are listed below:

**Exclusion criteria**

- Aged < 6 or >59 months
- Weight less than 4.5 Kgs.
- Enrolled in IPTi trial
- Severe malnutrition (defined as a child whose weight-for-height is below 3 standard deviations of less than 70% of the median of WHO reference values, or who has symmetrical edema involving at least the feet)
- No slide confirmed infection with *P. falciparum* or a mixed infection that includes a non-*P. falciparum* species
- Initial parasite density < 2,000 or > 200,000 asexual parasites per microliter.
- Presence of general danger signs among children < 5 years (inability to drink or breastfeed; vomiting everything; recent history of convulsions; lethargy or unconsciousness; inability to sit or stand up) or other signs of severe and complicated falciparum malaria according to WHO definitions
- Measured axillary temperature <37.5 °C
- Inability to attend stipulated follow-up visits
- Unwilling to provide informed consent provided by parent/guardian
- History of hypersensitivity reactions to any of the drugs being evaluated

Physician investigators will examine these patients to ascertain that they fit the above criteria. Patients with any of the above exclusion characteristics or who are for any other reason determined to be too ill for inclusion in the study, or who decline to participate in the study, will receive standard and appropriate treatment, according to MOH guidelines.

Standard recommended treatment doses of drugs included in the IPTi trial will be given orally depending on group assignment. The study will be conducted in an open-label (unblinded) fashion. SP will be studied first. Once SP recruitment is completed children will receive CD. All subjects will be observed for at least half an hour to monitor for adverse reactions and to make sure that the medicine is not vomited. If vomiting occurs within 30 min, a full dose will be re-administered and if vomiting occurs between 30 min and 1 hour, half the dose will be re-administered. If a child vomits a second time they will be excluded from the study and given rescue treatment (see IPTi Efficacy study- study drug doses and rescue treatment). All drug doses will be observed. Children with vomiting who appear dehydrated will be re-hydrated with oral rehydration solution or intravenous fluids depending on the judgment of the clinical staff.

**History and physical data**. Symptoms, prior anti-malarial drug use and physical examination data will be recorded on standard data forms– also known as a Case Record Form or CRF.

**Sample collection*. .*** Subjects will have a single finger prick.Samples will be collected for 1) Rapid diagnostic test for malaria, 2)Malaria blood films (one thick film and one thin film), 3) blood-onto-filter paper samples and a 4) haemoglobin estimation using a Hemocue at enrolment. Blood films will be Giemsa-stained and read for initial parasitemia screening, parasite speciation, and quantification. Filter papers will be air-dried and stored for analysis of *P. falciparum* genetic markers for drug resistance.

**Follow up*.*** With enrollment occurring on day 0, subjects will be followed actively on days 1, 2, 3, 7, 14, 21, and 28 by invitation to report to the clinic, and passively by 24-hour availability of a study clinician to evaluate and treat study subjects. At all active and passive follow-up times malaria blood films and filter paper strips will be obtained and brief history and physical evaluations made to seek signs or symptoms of persistent or recrudescent malaria. Filter papers will be used to look for parasite (*P. falciparum*) mutations linked to drug resistance and to examine parasite genotype with the aim of distinguishing recrudescence from re-infection.

A study clinician will promptly evaluate cases of post- treatment persistent malaria symptoms and parasitemia. Because parasitemia and the level of symptoms can rise transiently or persist for 2-3 days after treatment, study clinicians will look for danger signs as per the World Health Organization’s (WHO) definitions in determining whether to re-treat with anti-malarial drugs or provide symptomatic treatment during the first three days after treatment. After day 3, all cases of symptomatic parasitemia will be treated with a second or third line antimalarial drug according to local recommendations. Cases of severe malaria in any subject evaluated at any time, including the first three days after treatment and whether or not they are enrolled in the study, will be fully evaluated and admitted to hospital for appropriate medical care.

**Data analysis and interpretation**

*In vivo* data interpretation will be done using the standard WHO protocol for 28 days [35]. Treatment outcome will be categorized as adequate clinical and parasitological response (ACPR), early treatment failure (ETF), and late treatment failure (LTF) (late clinical failure [LCF] and late parasitological failure [LPF]). Results will be corrected to account for re-infection vs. recrudescence using PCR.

Clinical /Parasitological Outcomes (WHO 2003)

*Early Treatment Failure (ETF)*

- Development of danger signs or severe malaria on Day 1, 2, or 3, in the presence of parasitemia
- Parasitemia on Day 2 higher than Day 0 count irrespective of axillary temperature
- Parasitemia on Day 3 with axillary temperature ≥37.5°C
- Parasitemia on Day 3 ≥ 25% of count on Day 0

*Late Clinical Failure (LCF)*

- Development of danger signs or severe malaria from Day 4 to Day 28 in the presence of parasitemia, without previously meeting any of the criteria of ETF
- Presence of parasitemia and axillary temperature ≥37.5° C on any day from Day 4 to Day 28, without previously meeting any of the criteria of ETF

*Late parasitological failure (LPF)* –

Presence of parasitemia on Day 14, 21, or 28 and axillary temperature <37.5°C without previously meeting any of the criteria of ETF or LCF (after exclusion of re-infection by PCR for failures at days 14-28)

*Adequate Clinical and Parasitological Response (ACPR)* –

Absence of parasitemia on Day 28 irrespective of axillary temperature, without previously meeting any of the criteria of ETF, LCF or LPF

All day 0 samples from cases meeting criteria for resistance or treatment failure will be analyzed for molecular markers of antimalarial drug resistance as will the sample from the day that resistance was identified. In addition, two baseline sensitive/adequate clinical response samples will be randomly chosen as unmatched controls for each case of resistance or treatment failure in order to compare the prevalence of these molecular markers.
